# Maternal Body Weight and Gestational Diabetes Differentially Influence Placental and Pregnancy Outcomes

**DOI:** 10.1210/jc.2015-2590

**Published:** 2015-10-29

**Authors:** J. Martino, S. Sebert, M. T. Segura, L. García-Valdés, J. Florido, M. C. Padilla, A. Marcos, R. Rueda, H. J. McArdle, H. Budge, M. E. Symonds, C. Campoy

**Affiliations:** Early Life Research Unit (J.M., S.S., H.B., M.E.S.), Division of Child Health and Obstetrics & Gynaecology, School of Medicine, University of Nottingham, Nottingham NG7 2UH, United Kingdom; EURISTIKOS Excellence Centre for Paediatric Research (J.M., M.T.S., L.G.-V., C.C.), University of Granada, 18016 Granada, Spain; Department of Obstetrics and Gynaecology (J.F., M.C.P.), University of Granada, Granada, Spain; Immunonutrition Research Group (A.M.), Department of Metabolism and Nutrition, Institute of Food Science and Technology and Nutrition, Spanish National Research Council, E-28040 Madrid, Spain; Abbott Nutrition (R.R.), 18004 Granada, Spain; The Rowett Institute of Nutrition and Health (H.J.M.), University of Bucksburn, Aberdeen, AB21 9SB,United Kingdom; Institute of Health Sciences and Biocenter Oulu (S.S.), University of Oulu, 90014 Oulu, Finland

## Abstract

**Context:**

Maternal obesity and gestational diabetes mellitus (GDM) can both contribute to adverse neonatal outcomes. The extent to which this may be mediated by differences in placental metabolism and nutrient transport remains to be determined.

**Objective:**

Our objective was to examine whether raised maternal body mass index (BMI) and/or GDM contributed to a resetting of the expression of genes within the placenta that are involved in energy sensing, oxidative stress, inflammation, and metabolic pathways.

**Methods:**

Pregnant women from Spain were recruited as part of the “Study of Maternal Nutrition and Genetics on the Foetal Adiposity Programming” survey at the first antenatal visit (12–20 weeks of gestation) and stratified according to prepregnancy BMI and the incidence of GDM. At delivery, placenta and cord blood were sampled and newborn anthropometry measured.

**Results:**

Obese women with GDM had higher estimated fetal weight at 34 gestational weeks and a greater risk of preterm deliveries and cesarean section. Birth weight was unaffected by BMI or GDM; however, women who were obese with normal glucose tolerance had increased placental weight and higher plasma glucose and leptin at term. Gene expression for markers of placental energy sensing and oxidative stress, were primarily affected by maternal obesity as *mTOR* was reduced, whereas *SIRT-1* and *UCP2* were both upregulated. In placenta from obese women with GDM, gene expression for *AMPK* was also reduced, whereas the downstream regulator of mTOR, *p70S6KB1* was raised.

**Conclusions:**

Placental gene expression is sensitive to both maternal obesity and GDM which both impact on energy sensing and could modulate the effect of either raised maternal BMI or GDM on birth weight.

Obesity is of great importance to individual and global health (1). Its prevalence among women of reproductive age is increasing (2) so that, in Spain for example, up to 17% of pregnant women are obese (3). The increased prevalence of obesity in pregnant women has occurred concurrently with an increase in gestational diabetes mellitus (GDM) (4), which now affects up to 14% of all pregnancies in the United States, and around 2–6% of pregnancies in Europe (5, 6). Raised maternal body mass index (BMI) and GDM are both associated with adverse metabolic adaptations in the mother. These include increased risks of miscarriage and stillbirth, preeclampsia (7), and both intrauterine growth restriction and macrosomia (8), conditions with the potential to compromise fetal and newborn survival and health (9–11).

Consumption of an unhealthy diet in pregnancy has been linked to increased gestational weight gain (GWG) (12), BMI (13), and GDM (11), which are associated with fetal overgrowth (14). Placental nutrient supply is one mechanism linking maternal nutritional status and fetal growth and is dependent on utero-placental blood flow, hormone production, and nutrient transfer capacity, which is itself dependent on the type, number, and activity of a range of nutrient transporters (15). Increased glucose and lipid transport in GDM (16, 17) are also accompanied by placental defects arising from compromised trophoblast invasion and blood vessel formation (18). Although the association between high prepregnancy BMI and fetal overgrowth is well-established for type 1 diabetes (19), the effect of maternal BMI on placental function in women without GDM, its relationship to GWG (20), and its relationship to current diet remains unknown (21, 22).

Obesity is associated with perturbed maternal metabolism, raised plasma hormones, including leptin, insulin, and IGF 1, and the accumulation of inflammatory markers (eg, IL-6) (21). Insulin signaling is crucial for the regulation of intracellular and blood glucose concentrations. Alterations in the number of insulin-binding sites, reflecting placental insulin receptor expression, have been demonstrated in obesity (23) and diabetes mellitus (24). Fetal glucose, amino acids, and placental insulin/IGF 1 signaling act as upstream regulators of the mammalian target of rapamycin (mTOR), which is central to energy sensing and can be reset by maternal obesity and GDM (25) through phosphorylation mechanisms. These responses are mediated through changes in nuclear factor κB signaling, thereby resetting proinflammatory and pro-oxidative pathways (26) acting through Toll-like receptor 4 (TLR4) (27). Furthermore, mTOR inactivation occurs through the AMP-activated protein kinase (AMPK) pathway (28), whereas uncoupling protein (UCP)-2 limits oxidative damage within the placenta by decreasing reactive oxygen species (ROS) production (29). Free fatty acids also decrease peroxisome proliferator-activated receptor (PPAR)-γ expression (30) while activating myeloid proinflammatory cells, although whether these placental responses can be modulated by BMI and/or GDM is not established.

In the present study, we aimed to determine whether maternal BMI and/or GDM influenced placental homeostasis and energy balance and thus affect birth outcomes. The establishment of direct links between maternal nutritional status, the placenta, and weight at birth will give insight on mechanistic pathways, thereby enabling targeted interventions designed to prevent adverse outcomes under these conditions.

## Materials and Methods

### Participants

The subjects participated in a Spanish longitudinal study on the influence of body composition by maternal genetics and nutrition (“Study of Maternal Nutrition and Genetics on the Foetal Adiposity Programming” [PREOBE] study: P06-CTS-02341) undertaken between 2007 and 2010 and registered with www.ClinicalTrials.gov, (NCT01634464) (31, 32). It was conducted according to the guidelines in the Declaration of Helsinki, and all experimental procedures approved by the Ethics Committees for Granada University, San Cecilio University Hospital, and the University of Nottingham. Witnessed, written informed consent was obtained from all subjects before their study inclusion, and participants were assured of anonymity. Anthropometric assessments of were undertaken following the standards established by the Spanish Society of Gynaecology and Obstetrics, the Fetal Foundation, and the Spanish Association of Pediatrics.

**Figure 1.**
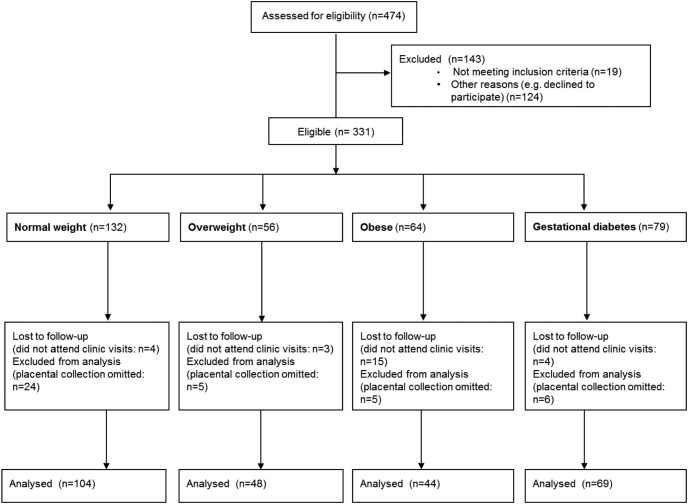
Participants in the Study of Maternal Nutrition and Genetics on the Foetal Adiposity Programming and classification following body mass index and gestational diabetes mellitus criteria.

In the overall PREOBE study (Figure 1), 474 pregnant women aged 18–45, with singleton pregnancies, were assessed for eligibility between 12–20 weeks' gestation at two different primary health care settings (Clinical University Hospital “San Cecilio” and the “Mother-Infant” University Hospital) in Granada, Spain. Among these, 124 declined to participate. Criteria for exclusion (n = 19) were participation in another study simultaneously, receiving drug treatments, being underweight (BMI <18.5 kg/m^2^), or having type 1 diabetes or preexisting disease. Therefore, 331 women were included in the project and classified according to their BMI (based on self-reported prepregnancy weight provided on enrollment) as normal weight (prepregnancy BMI ≥18.5 but <25 kg/m^2^; n = 132), overweight (prepregnancy BMI ≥25 but <30 kg/m^2^; n = 56), and obese (prepregnancy BMI ≥30 kg/m^2^; n = 64). In addition, 79 women were diagnosed with GDM following measurement of raised fasting plasma glucose concentrations, 25 women after a 75-g oral glucose tolerance test between 16 and 18 weeks' gestation (11), if they either had a family history of GDM or had previously had GDM, or were obese, whereas 54 women were diagnosed after an additional 100-g oral glucose tolerance test between 24 and 28 weeks' gestation.

The number of women in each BMI group for whom collection of biological samples was achieved at the time of delivery is shown in Figure 1. Among these, a subpopulation of 135 subjects underwent molecular analysis in Nottingham (ie, approximately half of those sampled within each group: 59 normal weight, 29 overweight, 22 obese, 25 GDM). The 25 mothers with GDM were subsequently classified according to their BMI as normal weight GDM (prepregnancy BMI ≥18.5 but <25 kg/m^2^; n = 14) and obese GDM (prepregnancy BMI ≥30 kg/m^2^; n = 11). Participants diagnosed with GDM then had increased medical supervision and received nutritional advice for meal plans designed to control normoglycemia, with none receiving insulin.

During pregnancy, each mother attended additional PREOBE study medical visits at 24 (BMI group) or 34 weeks of gestation (BMI and GDM groups), Gestational age was calculated from the last menstrual period and through ultrasound scan, considering a gestational age below 37 weeks as preterm delivery. Anthropometric characteristics of the fetus were estimated by using ultrasound scan at 34 gestational weeks. When there was a disagreement between the last menstrual period and ultrasound, the measurements taken by ultrasound were used to calculate the gestational age (33).

Maternal weight gain (GWG) during pregnancy was defined as weight change to the last recorded weight in the 34th gestational week and compared to the 2009 Institute of Medicine (IOM) guidelines (34). Large (LGA) and small (SGA) for gestational age infants were defined according to the Lubchenco growth curves (35) with standard adjustment for gestational age at birth (ie, birth weights greater than the 90th population centile were defined as LGA infants; those less than the 10th population centile as SGA).

### Maternal nutrient intake

Nutrient intake was collected during late gestation (34–40 weeks) using standardized 7-day dietary records given during the patients' second visit. Each participant was given verbal and written instructions on how to record food and drinks consumed with a booklet of common food items and mixed dishes to facilitate estimation of portion sizes. Near delivery, food records were reviewed individually by a nutritionist for completeness and accuracy of food description and portion size. Nutritional data were analyzed for nutrient intake by using a nutritional software program (CESNID 1.0, Barcelona University) based on validated Spanish food tables (36). These results were compared with a food frequency questionnaire taken at 24 weeks' gestation, and both sets of records were reviewed with the mother around the time of delivery by a professional nutritionist with respect to their accuracy, thereby avoiding the potential inaccuracies associated with these types of records (37).

### Collection and analysis of blood samples

Maternal venous blood was collected at 24 and 34 weeks of gestation and during labor. Umbilical venous blood samples were collected within 30 minutes after placental delivery from a double-clamped section of umbilical cord. EDTA and serum collection tubes were used (Vacutainer: 368857 and 367953) for hematological assessment and biochemical analyses respectively. Blood samples for serum preparation were left at 4 C for 15 minutes to allow blood clotting, centrifuged at 3500 rpm for 10 minutes, and the serum fraction transferred into sterile tubes. Samples were stored at 4 C for same-day analyses or at –80 C for further analysis. Hematological parameters were analyzed using a hematology analyzer (Sysmec XE-2100, Roche Diagnostics) and flow cytometer (Advia 120-160858, Bayer HealthCare). Plasma glucose and triglycerides were measured enzymatically (Modular Analytics EVO, Roche), whereas serum leptin concentrations measured by ELISA (Biosource Kap 2281).

### Collection of placenta samples

Placenta were collected and weighed immediately after delivery. Disc samples containing both maternal and fetal tissue were obtained from identical portions of the placental plate to avoid any regional variations. Visual inspection of the placenta for necrosis or any other abnormality was undertaken by experienced clinicians. This included the measurement of placental size, weight, and morphology; if there was any abnormality such as multilobules or placenta spuria, annular, membranous, infarction, chorangiosis, or vasculopathies, a sample was obtained from a healthy region. Then, after removal of the decidua, a representative 0.5 × 0.5 × 0.5 cm (200 mg) sample was excised from the middle of the radius (distance between the insertion of the umbilical cord and the periphery) of each placenta, rinsed twice with saline solution (NaCl 0.9%), and immediately placed into sterile 1.5-ml microtubes containing RNAlater solution (Qiagen Ltd). All samples were stored under RNase free conditions using liquid nitrogen before storage at –80 C for later analysis in Nottingham.

### Laboratory analysis

#### Gene expression

Total RNA was extracted from 100 mg of maternal placenta tissue using 200 μl of chloroform per 1 mL of TRI reagent solution (Sigma Chemical Co. Poole, UK) and RNeasy extraction kit (Qiagen Ltd). A total of 2 μg RNA was used to generate 20 μl cDNA using a High Capacity RNA-to-cDNA kit (Applied Biosystems). Negative control RT samples lacking Enzyme Mix (-RT) were included for each sample.

Real-time PCR using 15 μl of reactions consisting of 4.5 μl diluted 1:10 cDNA, 3.0 μl (final concentration of 250 nM) gene-specific primers (Table 1), and 7.5 μl of SYBR Green mastermix (Thermo Scientific, ABgene Ltd) were performed. Duplicate samples were run for 40 cycles with negative controls in 96-well plates using the Techne Quantica Thermocycler (Techne Inc., Barloword Scientific). Ten-fold serial dilutions of cDNA for each gene were used to generate standard curve analysis and only experiments with r^2^ > 0.985 were included. Cycle threshold measurements, calculated by 2^−ΔCt^ method (38), were used for mRNA expression. Human 18S rRNA was used as a housekeeping gene for data normalization.

**Table 1.** Sociodemographic Characteristics and Birth Weights of All Participants, With and Without GDM: N, OW, O, GDMN, and GDMO Pregnant Women

Maternal Characteristics	N (n = 59)	OW (n = 29)	O (n = 22)	GDMN (n = 14)	GDMO (n = 11)
Age at delivery (y)	30.4 ± 4.5	30.9 ± 7.2	29.0 ± 4.7	33.1 ± 4.1^a^	34.7 ± 4.3^b^
Unemployed (%)	32.2	28.6	38.1	14.3	66.7^a^
Higher education (university) (%)	42.4	42.9	22.7	42.8	10
Smoking during pregnancy (%)	12.1	25^a^	9.1	0	0
Primiparous (%)	58.6	46.4	63.6	57.1	60
Height (cm)	162.9 ± 5.7	162.5 ± 6.4	162.7 ± 6.2	159.3 ± 3.9	160.5 ± 6.0
Pregestational BMI (kg/m^2^)	21.8 ± 1.8	27.8 ± 2.2^c^	32.5 ± 2.6^c^	22.4 ± 1.8	35.5 ± 4.9^c^
BMI at 34 weeks (kg/m^2^)	26.6 ± 2.6	31.3 ± 2.4^c^	35.4 ± 2.4^c^	25.9 ± 2.6	36.4 ± 4.1^c^
GWG 0–34 weeks (kg) (32)	12.6 ± 4.3	9.9 ± 4.6^b^	7.3 ± 5.1^c^	9.0 ± 5.6^b^	2.2 ± 7.8^c^
LGWG (kg and % of women in BMI category: n = 15, 5, 8, 8, and 6, respectively)	7.3 ± 1.9 (25%)	2.7 ± 1.9^c^ (18%)	2.5 ± 1.7^c^ (36%)	5.2 ± 3.5^a^ (57%)	−3.4 ± 5.0^c^ (55%)
AGWG (kg and % of women in BMI category: n = 23, 9, 5, 3, and 2, respectively)	12.1 ± 1.1 (39%)	8.2 ± 1.2^c^ (32%)	5.9 ± 0.9^c^ (23%)	11.4 ± 1.0 (22%)	5.2 ± 0.6^c^ (18%)
HGWG (kg and % of women in BMI category: n = 21, 14, 9, 3, and 3, respectively)	17.0 ± 2.9 (36%)	13.6 ± 2.4^b^ (50%)	12.3 ± 3.4^b^ (41%)	17.0 ± 1.1 (21%)	11.3 ± 3.2^b^ (27%)
BW for each GWG 0–34 weeks (g) (32)					
LGWG (n = 15, 5, 8, 8, and 6, respectively)	3410 ± 116	3496 ± 241	3253 ± 158	3307 ± 151	3373 ± 221
AGWG (n = 23, 9, 5, 3, and 2, respectively)	3160 ± 61	2870 ± 109	3318 ± 274	3493 ± 334	3090 ± 350
HGWG (n = 21, 14, 9, 3, and 3, respectively)	3348 ± 101	3475 ± 117	3707 ± 156	3433 ± 98	3716 ± 301
No. of cesarean deliveries (%)	12.3	25.9	38.1	25	50^a^
Preterm delivery (<37 gestational weeks) (%)	3.4	3.4	9.1	14.3	27.3^a^
Male newborn (%)	52.5	39.3	61.9	57.1	72.7

Abbreviations: AGWG, adequate gestational weight gain; BMI, body mass index; GDM, gestational diabetes mellitus; GDMN, gestational diabetic normal weight; GDMO, gestational diabetic mellitus obese; GW, gestational weight; GWG, gestational weight gain; LGWG, low gestational weight gain; N, normal weight; O, obese; OW, overweight.

Values are means ± sd or categorical data as appropriate. GWG during the first 34 gestational weeks based on 2009 Institute of Medicine guidelines for each category (32). LGWG was classified as low: <9.8 kg for normal weight, <5.9 kg for overweight, and <4.2 kg for obese women. AGWG was classified as adequate: 9.8–13.6 kg for normal weight, 5.9–9.8 kg for overweight, and 4.2–7.6 kg for obese women. HGWG was classified as high: >13.6 kg for normal weight, >9.8 kg for overweight, and >7.6 kg for obese women.

Statistical differences: ^a^ *P* < 0.05,

b *P* < 0.01,

c *P* < 0.001 compared to normal weight group (χ^2^ test or *t* independent test for continuous variables; χ^2^ test for categorical variables).

### Placental triglyceride and thiobarbituric active reactive substance content

Total lipid extraction used an adapted Folch method and the triglyceride concentration, determined spectrophotometrically (Randox Laboratories Ltd). Thiobarbituric active reactive substance (TBARS) was determined as described by Mistry et al (39).

### Statistical analysis

Analyses were performed using IBM SPSS, v20.0, statistical software for Windows (IBM Corp). To assess the data for normality, a Kolmogorov–Smirnov test was performed, where a *P* value >.05 indicated normal distribution. Thereafter, appropriate parametric, or nonparametric, tests were used to analyze the effects of maternal overweight and obesity as follows: 1) anthropometrical and physiological comparisons among comparable groups of mothers, placentas, and newborns were made using a Student's *t* test between relevant groups and 2) comparisons of gene expression were determined by using Mann-Whitney test. Categorical data were analyzed using χ^2^ test of independence. The study was not designed to look at the effect of fetal gender on the placenta. Continuous data presented are expressed as a mean average with their standard errors (SEM), with *P* value <.05 deemed to represent statistical significance.

## Results

### Maternal characteristics, pregnancy outcome, placental composition, and metabolic status

Obese women with GDM were older, more likely to be unemployed, and to have lower educational attainment. Women with obesity gained less weight up to 34 weeks' gestation compared to those of normal weight and glucose tolerance (Table 1). In particular, obese women with GDM gained significantly less weight than the 2009 IOM guidelines for their BMI group (χ^2^ test, *P* = .04) and reflected their lower total energy and carbohydrate intake (Table 2). They also had a lower lipid intake primarily as a consequence of decreased saturated fatty acid consumption. The importance of IOM-classified GWG (34) on birth weight was reflected in the trend for obese women to deliver bigger infants when gaining more weight than recommended (Table 1).

**Table 2.** Maternal Energy and Nutrient Intake: N, OW, O, GDMN, and GDMO Pregnant Women

Maternal Dietary Intake	N (n = 37)	OW (n = 15)	O (n = 8)	GDMN (n = 11)	GDMO (n = 6)
Energy (kcal)	2155 ± 339	2114 ± 784	1831 ± 560^a^	1879 ± 379^a^	1656 ± 348^b^
Total carbohydrates (g)	237 ± 54	217 ± 63	189 ± 69^a^	187 ± 31^b^	173 ± 46^b^
Total proteins (g)	83.9 ± 17.5	84.5 ± 28.4	74.8 ± 1.2	84.4 ± 23.0	74.8 ± 11.9
Total lipids (g)	90.5 ± 19.4	95.6 ± 54.2	86.5 ± 26.4	81.7 ± 27.4	68.7 ± 14.7^a^
SFA (g)	33.8 ± 8.3	36.6 ± 25.9	30.3 ± 8.9	28.3 ± 13.8	21.4 ± 5.6^b^
MUFA (g)	36.2 ± 9.6	38.3 ± 19.2	35.1 ± 14.5	36.5 ± 12.1	31.8 ± 10.1
PUFA (g)	12.8 ± 4.3	12.5 ± 7.2	13.7 ± 4.6	10.1 ± 2.0	9.7 ± 2.7

Abbreviations: GDMN, gestational diabetic normal weight; GDMO, gestational diabetic mellitus obese; MUFA, monounsaturated fatty acid; N, normal weight; O, obese; OW, overweight; PUFA, polyunsaturated fatty acid; SFA, saturated fatty acid.

Values are means ± sd; n, number of women per group.

Statistical differences: ^a^ *P* < 0.05,

b *P* < 0.01 compared to normal weight group (*t* independent test for continuous variables).

A majority of women gave birth normally at term, with obese women with GDM having a greater risk of preterm delivery and cesarean section (Table 1). Although estimated fetal weight at 34 gestational weeks was higher when GDM was accompanied by obesity, size and weight at birth were not different between these groups (Table 3). The increased fetal weight at late gestation is likely to reflect the higher preterm and cesarean section delivery rate for obese women with GDM (Table 1). However, although maternal obesity alone did not affect size at birth, women who were obese with normal glucose tolerance had increased placental weight and LGA infants.

**Table 3.** Anthropometric and Clinical Characteristics of Infants Born to Mothers With and Without Gestational Diabetes: N, OW, O, GDMN, and GDMO Mothers

Infant Characteristics	N (n = 59)	OW (n = 29)	O (n = 22)	GDMN (n = 14)	GDMO (n = 11)
Estimated fetal weight at 34 weeks' gestation (g)	2363 ± 183	2345 ± 183	2393 ± 383	2467 ± 380	2541 ± 501^a^
Placental weight (g)	469 ± 120	495 ± 135	531 ± 114^a^	498 ± 134	476 ± 93
Placental to birth weight ratio	0.143 ± 0.031	0.157 ± 0.046	0.158 ± 0.041	0.147 ± 0.035	0.139 ± 0.017
Gestational age (weeks)	39.2 ± 1.0	39.4 ± 1.6	39.3 ± 1.7	39.3 ± 1.3	38.8 ± 1.3
Newborn length (cm)	50.2 ± 1.8	50.5 ± 1.5	50.6 ± 2.7	50.6 ± 1.7	50.9 ± 3.4
Newborn weight (g)	3292 ± 410	3230 ± 587	3454 ± 549	3374 ± 402	3415 ± 549
SGA (n) (%)	4 (6.8)	3 (10.3)	1 (4.5)	1 (7.1)	2 (18.2)
AGA (n) (%)	52 (88.1)	24 (82.8)	16 (72.8)	12 (85.8)	7 (63.6)
LGA (n) (%)	3 (5.1)	2 (6.9)	5 (22.7)^a^	1 (7.1)	2 (18.2)
Ponderal index (g/cm^3^ × 100)	2.62 ± 0.27	2.56 ± 0.49	2.58 ± 0.28	2.60 ± 0.34	2.60 ± 0.42

Abbreviations: AGA, average for gestational age (10% < birth weight population centile <90%); GDMN, gestational diabetic normal weight; GDMO, gestational diabetic mellitus obese; LGA, large for gestational age (birth weight population centile >90%); N, normal weight; O, obese; OW, overweight; SGA, small for gestational age (birth weight population centile <10%).

Values are means ± sd. Anthropometric characteristics of the fetus were estimated by using ultrasound scan at 34 gestational weeks.

Statistical differences: ^a^ *P* < 0.05 compared to normal weight group (*t* independent test for continuous variables; χ^2^ test for categorical variables).

Close to delivery, maternal blood glucose was elevated in women with GDM irrespective of BMI (Table 4). Triglyceride concentrations and monocyte counts were similar between groups, but monocyte count was higher in the cord blood of obese women with normal glucose tolerance. Serum leptin concentrations at delivery were elevated in obese compared to normal weight mothers and their offspring. Placental triglyceride content was raised in obese women with GDM but with no difference in TBARS.

**Table 4.** Maternal, Placental, and Cord Blood Metabolic Characteristics: N, OW, O, GDMN, and GDMO Pregnant Women

Maternal Blood at Term	N (n = 59)	OW (n = 29)	O (n = 22)	GDMN (n = 14)	GDMO (n = 11)
Glucose (mmol/liter)	4.3 ± 1.3	4.6 ± 1.3	5.3 ± 2.3^a^	6.0 ± 2.2^c^	6.1 ± 1.9^c^
Triglyceride (mmol/liter)	11.7 ± 3.9	13.2 ± 4.2	12.8 ± 4.3	11.6 ± 3.9	12.3 ± 3.3
Leptin (μg/liter)	16.0 ± 13.6	24.2 ± 23.5	33.9 ± 21.9^b^	21.1 ± 16.4	36.6 ± 19.9^b^
Monocyte count (×10^9^/liter)	0.5 ± 0.2	0.6 ± 0.2	0.6 ± 0.2	0.6 ± 0.3	0.40 ± 0.2

Cord Blood^d^	N (n = 33)	OW (n = 18)	O (n = 16)	GDN (n = 10)	GDO (n = 7)
Glucose (mmol/liter)	3.8 ± 1.2	3.6 ± 1.2	3.3 ± 1.3	3.9 ± 0.7	4.3 ± 1.0
Triglyceride (mmol/liter)	2.7 ± 1.1	2.5 ± 0.8	2.5 ± 1.4	2.5 ± 1.3	2.5 ± 1.2
Leptin (μg/liter)	19.7 ± 17.9	32.7 ± 19.6	62.3 ± 69.0^a^	42.6 ± 31.5^a^	36.6 ± 19.9
Monocyte count (×10^9^/liter)	1.0 ± 0.4	1.1 ± 0.4	1.4 ± 0.6^a^	1.2 ± 0.8	1.1 ± 0.4

Placental Tissue	N (n = 59)	OW (n = 29)	O (n = 22)	GDN (n = 14)	GDO (n = 11)
Total placental TG (mg/g) per placental weight (g)	19.9 ± 10.0	22.0 ± 10.9	23.9 ± 12.8	19.7 ± 10.6	28.3 ± 16.5^a^
Relative placental TBARS	1.0 ± 0.3	0.9 ± 0.3	0.8 ± 0.2	1.0 ± 0.3	1.0 ± 0.3

Abbreviations: GDMN, gestational diabetic normal weight; GDMO, gestational diabetic mellitus obese; N, normal weight; O, obese; OW, overweight; TBARS, thiobarbituric acid reactive substance; TG, triglyceride.

Values are means ± sd.

Statistical differences: ^a^ *P* < .05,

b *P* < .01, and ^c^ *P* < .001 compared to normal weight group (*t* independent test for continuous variables).

d See text for information on missing individuals.

### Maternal body weight, GDM, and placental markers of energy homeostasis, cell growth, and endocrine sensitivity

Maternal obesity was accompanied with reduced placental gene expression for *mTOR* (Table 5), whereas upstream (ie, *Akt*) and downstream (ie, *p70S6KB1*) signaling molecules for *mTOR* were unaffected. Placental mRNA abundance for *p70S6KB1* was increased when obesity was accompanied by GDM. In addition, GDM was associated with reduced placental gene expression for *AMPK* irrespective of BMI. Increased placental leptin gene expression in normal weight women with GDM was reversed when GDM was accompanied by obesity. There were no differences in *LEPR* gene expression between groups. Markers of oxidative stress (ie, *SIRT1* and *UCP2*) were up-regulated in overweight and obese women, but not by GDM. Placental gene expression for *GR*α increased with maternal GDM but was not affected by obesity, and no differences were apparent for inflammatory markers *PPAR*γ and *TLR4*, or indices of insulin action (ie, *IGF1R* or *IRS1*). There was no evidence of any effect of gestational age, mode of delivery, or insulin administration on any of these outcomes.

**Table 5.** Effects of Maternal BMI on Gene Expression Markers of Energy Sensing and Balance, Oxidative Stress, and Inflammation in Placenta of N, OW, O, GDMN, and GDMO Pregnant Women

Pathway Gene	NCBI Sequence	Target Gene	N (n = 59)	OW (n = 29)	O (n = 21)	GDMN (n = 14)	GDMO (n = 11)
Energy sensing	NM_006251	*AMPK*	1.0 ± 0.1	0.9 ± 0.1	1.0 ± 0.2	0.6 ± 0.1^a^	0.4 ± 0.1^c^
	NM_001014432	*Akt1*	1.0 ± 0.1	1.0 ± 0.2	1.2 ± 0.2	0.8 ± 0.1	0.9 ± 0.1
	NM_004958	*mTOR*	1.0 ± 0.1	0.7 ± 0.1	0.5 ± 0.1^a^	0.6 ± 0.1	0.5 ± 0.1
	NM_003161	*p70S6KB1*	1.0 ± 0.1	1.1 ± 0.2	1.6 ± 0.4	0.7 ± 0.2	1.4 ± 0.2^a^
Energy balance	NM_000230	*LEP*	1.0 ± 0.2	1.5 ± 0.5	0.9 ± 0.4	4.1 ± 1.1^a^	0.8 ± 0.4
	NM_002303	*LEPR*	1.0 ± 0.2	0.8 ± 0.1	1.1 ± 0.3	0.5 ± 0.0	0.5 ± 0.1
Insulin action	NM_000875	*IGF1R*	1.0 ± 0.1	1.2 ± 0.1	1.2 ± 0.2	1.0 ± 0.1	0.8 ± 0.1
	NM_005544	*IRS1*	1.0 ± 0.1	1.1 ± 0.1	1.4 ± 0.2	0.9 ± 0.1	0.8 ± 0.1
Oxidative stress	NM_001033611.1	*UCP2*	1.0 ± 0.2	1.4 ± 0.2^b^	1.4 ± 0.2^a^	1.3 ± 0.4	0.8 ± 0.3
	NM_001142498	*SIRT1*	1.0 ± 0.1	1.4 ± 0.2^a^	1.6 ± 0.2^c^	0.8 ± 0.2	1.5 ± 0.3
Inflammation	NM_015869.4	*PPAR-*γ	1.0 ± 0.1	0.9 ± 0.1	0.9 ± 0.1	1.0 ± 0.2	0.9 ± 0.1
	NM_001135930.1	*TLR4*	1.0 ± 0.1	0.9 ± 0.1	0.9 ± 0.1	0.8 ± 0.1	0.8 ± 0.2
	NM_000176	*GR-*α	1.0 ± 0.1	1.2 ± 0.1	1.0 ± 0.1	1.2 ± 0.1^a^	1.5 ± 0.2^a^

Abbreviations: BMI, body mass index; GDMN, gestational diabetic normal weight; GDMO, gestational diabetic mellitus obese; N, normal weight; NCBI, National Center for Biotechnology Information; O, obese; OW, overweight.

Data are nonparametric and represent mean ± sd. Data expressed relative to housekeeping gene (18S rRNA), normalized to the control group to give the fold change.

Statistical differences: ^a^ *P* < .05,

b *P* < .01, and ^c^ *P* < .001 compared to normal weight (Mann-Whitney test).

## Discussion

Our major finding is the differential effects of perturbations in energy homeostasis on placental expression of genes regulating placental size, function, and endocrine sensitivity with raised BMI and GDM. Maternal obesity, but not GDM, contributed to greater placental weight, whereas placental adaptation was demonstrated in markers of energy sensing for both groups. Reduced placental *AMPK* mRNA expression with GDM but not with obesity alone and suppression of gene expression for *mTOR* with obesity are indicative of complementary control mechanisms. Furthermore, the *mTOR* downstream regulator, *p70S6KB1*, was increased by obesity even without GDM. Consequently, as maternal glucose was raised at term, and with GDM, these responses could be mediated by changes in glucose homeostasis (28, 40).

Surprisingly, placental gene expression for *IRS1* and *IGFR1* was not affected by obesity or GDM, findings that differ with those described by Jansson et al (41) in a cohort of Swedish women, in which placental activation of *mTOR* was accompanied by enhanced insulin/IGF 1 signaling with raised BMI. However, there are important demographic differences between studies, because the obese Swedish women had a higher mean BMI and substantially greater GWG than our Spanish women. Therefore, the discrepancy between studies may reflect placental threshold effects in response to excess energy intake (42, 43). In the overweight and obese PREOBE women studied here, reduced placental *mTOR* gene expression was accompanied with raised *SIRT1* and *UCP2*, suggesting enhanced antioxidant capacity (44). These findings indicate an adaptive placental response to increased BMI, in line with the physiological role of mitochondria in regulating cellular ATP and AMP concentrations (45). This could occur through changes in the activity of AMPK, Akt, and mTOR, with the former sensing energy depletion (46) and the latter stimulated by raised energy supply (43). Mitochondria also regulate ROS production and oxidative stress by uncoupling energy supply, with both *AMPK* and *mTOR* modulating oxidative stress through changes in UCP2 (47) and nuclear factor κB action (26, 48), thereby promoting proinflammatory and pro-oxidative pathways within trophoblast cells. In contrast, mitochondrial replication is dependent on *SIRT1* activity that also determines cell survival and senescence by inhibiting *mTOR* activity (49).Our findings are, therefore, indicative of a protective or physiological adaptation by the placenta against oxidative stress (49, 50) with raised maternal BMI. This is further supported by the stability of placental TBARS content, a marker of oxidative stress (44), between groups, suggesting that the fetus is protected from excess ROS. These responses were accompanied by similar expression of placental genes involved in inflammatory responses, such as *PPAR*α (30) and *TLR4* (27), suggesting inflammation was not directly promoted with raised BMI (30).

Although there were no differences in maternal triglyceride concentrations, obesity with GDM leads to placental triglyceride accumulation, which (51) has been shown to be correlated with fetal adiposity (52) reflected in the increase in LGA infants with maternal obesity. Increased placental triglyceride storage with GDM was accompanied by up-regulation of placental GRα that has been shown in an ovine model on nutritional manipulation of placental growth to follow changes in placental mass with gestation (53).

As expected, maternal obesity was associated with higher plasma leptin irrespective of GDM although whether this leads to a direct inhibitory effect on food intake (54) as reported by these women or reflects maternal metabolism complicated by leptin resistance (55) is uncertain. Although the placenta is a source of plasma leptin (56), which can be stimulated by obesity and GDM (57, 58), we did not observe differences in leptin gene expression, suggesting that adipocytes, rather than the placenta, are the main origin of differences in plasma leptin (59). An alternative explanation is that there are changes in leptin turnover or that leptin regulated its own expression within the placenta through a mechanism involving the suppression of AMPK (60). Effects on placental leptin expression through the action of glucocorticoids has also been described (61), and is compatible with our observations of an increase in placental GRα, suggesting a local inflammatory response within the placenta of obese gestational diabetic women (62, 63).

Plasma leptin concentrations were raised in cord blood of infants born to obese and obese GDM mothers. This could reflect increased transplacental substrate supply from raised maternal plasma glucose in these women acting through fetal insulin to then promote fetal fat deposition (11, 64). An enhanced glucose–insulin pathway can promote offspring adiposity (11), whereas the adipokine leptin stimulates cell proliferation by inducing the IRS1/MAPK pathway in a glucose-dependent manner (65). Furthermore, although fetal hyperleptinemia can contribute to inducing leptin resistance by chronic activation of leptin receptors in the fetus (66), it is not known whether hypothalamic leptin targets are responsive before birth or whether neonatal leptin resistance leads to long-term adverse consequences. Enhanced circulating leptin in obese women was associated with higher leptin and monocyte concentrations in cord blood. In addition to its potential role in newborn adiposity (64, 67), growing evidence has linked leptin with the maturation of the hypothalamus (68) and the fetal and neonatal immune system (69), leading to impaired immune responses (59). As part of the PREOBE follow-up, further studies are exploring potential long-term implications of obesity and diabetes in offspring neurodevelopment through functional measurements. This will enable a more direct assessment of any effect on differences in leptin surge between infants born into the study and their subsequent brain development. Increased proinflammatory cytokine expression, including tumor necrosis factor-α and IL-6, and/or enhanced circulating monocyte chemo-attractant protein 1 concentrations in obese women may account for raised monocytes concentrations in cord blood of their infants (70). Higher plasma monocyte chemo-attractant protein 1 (71) has been implicated in monocyte recruitment into adipose tissue of newborns from obese individuals (70) and ultimately produce proinflammatory cytokines, contributing to a state of insulin resistance and low-grade inflammation.

As the relative risk of obese and GDM women producing an LGA infant is substantial (11, 14, 72); one strategy to prevent this outcome (73) is through healthier food choices (74). In our study, the first line of treatment of GDM was through nutrition and lifestyle advice in maternity welfare clinics. These reinforced local secular food preferences of Spanish women of primarily Hispanic European white origin (95–98%) for a Mediterranean diet rich in polyunsaturated fatty acids, fruits, and vegetables (75, 76), which contrasts with those of Northern European and American women recruited in previous studies (77, 78). However, although there was no difference in mean birth weight in our study, maternal obesity was associated with a higher incidence of LGA infants despite lower self-reported energy and macronutrient intakes. The latter may reflect recall bias because women with increased BMI do not always accurately report their food intake (79, 80). Alternatively, nutrient supply to the fetus of obese woman may be more dependent on existing maternal nutrient stores and current metabolic state (81) than daily intakes. This is supported by raised plasma glucose concentrations even in those obese women who were not diagnosed with GDM. Furthermore, the dietary advice given to these women, despite lowering GWG, did not reduce the incidence of LGA infants, although it is acknowledged that the study was not powered to directly assess such an outcome.

In conclusion, placental gene expression is sensitive to both maternal BMI and GDM, which affects both placental triglyceride content and energy sensing. These adaptations could modulate maternal and fetal glucose homeostasis and thus prevent some of the potential adverse consequences on fetal growth and body composition.
